# Histochemical Evidence for Reduced Immune Response in Nasal Mucosa of Patients with COVID-19

**DOI:** 10.3390/ijms25084427

**Published:** 2024-04-17

**Authors:** Nicole Power Guerra, Martin Bierkämper, Jessica Pablik, Thomas Hummel, Martin Witt

**Affiliations:** 1 Smell & Taste Clinic, Department of Otorhinolaryngology, Faculty of Medicine Carl Gustav Carus, Technische Universität Dresden, 01309 Dresden, Germany; nicole.powerguerra@ukdd.de (N.P.G.); martin.bierkaemper@mailbox.tu-dresden.de (M.B.); thomas.hummel@tu-dresden.de (T.H.); 2 Department of Pathology, Faculty of Medicine Carl Gustav Carus, Technische Universität Dresden, 01309 Dresden, Germany; jessica.pablik@ukdd.de; 3 Department of Anatomy, Institute of Biostructural Foundations of Medical Sciences, Poznań University of Medical Sciences, 61-781 Poznań, Poland; 4 Department of Anatomy, Faculty of Medicine Carl Gustav Carus, Technische Universität Dresden, 01309 Dresden, Germany

**Keywords:** COVID-19, immunohistochemistry, immune response, SARS spike protein, post mortem, T cells

## Abstract

The primary entry point of severe acute respiratory syndrome coronavirus 2 (SARS-CoV-2) is the nasal mucosa, where viral-induced inflammation occurs. When the immune response fails against SARS-CoV-2, understanding the altered response becomes crucial. This study aimed to compare SARS-CoV-2 immunological responses in the olfactory and respiratory mucosa by focusing on epithelia and nerves. Between 2020 and 2022, we obtained post mortem tissues from the olfactory cleft from 10 patients with histologically intact olfactory epithelia (OE) who died with or from COVID-19, along with four age-matched controls. These tissues were subjected to immunohistochemical reactions using antibodies against T cell antigens CD3, CD8, CD68, and SARS spike protein for viral evidence. Deceased patients with COVID-19 exhibited peripheral lymphopenia accompanied by a local decrease in CD3^+^ cells in the OE. However, SARS-CoV-2 spike protein was sparsely detectable in the OE. With regard to the involvement of nerve fibers, the present analysis suggested that SARS-CoV-2 did not significantly alter the immune response in olfactory or trigeminal fibers. On the other hand, SARS spike protein was detectable in both nerves. In summary, the post mortem investigation demonstrated a decreased T cell response in patients with COVID-19 and signs of SARS-CoV-2 presence in olfactory and trigeminal fibers.

## 1. Introduction

In early 2020, the identification of severe acute respiratory syndrome coronavirus type 2 (SARS-CoV-2) marked the onset of the COVID-19 pandemic [1]. During acute SARS-CoV-2 infection, approximately 80 % of the affected individuals experienced mild upper respiratory tract symptoms with or without fever. The remaining cases progressed to severe and/or critical pneumonia often resulting in acute respiratory distress syndrome or mortality [2,3,4]. The nasal cavity serves as the entry point, and the elevated viral load observed in this region constitutes the primary initial site of SARS-CoV-2 infection and the subsequent immune response [5,6,7]. The present study, however, focused on the cellular level of the immune response within the nasal cavity of deceased patients with COVID-19.

Coronaviruses, which are membrane-coated RNA viruses, exhibit virions with an extensive array of spike (S) proteins on their surface [8,9]. The S protein of SARS-CoV-2 utilizes the protease angiotensin-converting enzyme 2 (ACE-2) as a receptor for entry into human host cells, facilitated by other proteases like TMPRSS2 [10,11]. The efficient replication and excretion of SARS-CoV-2 from the upper respiratory tract can be explained by the high levels of co-expression of ACE-2 and TMPRSS2 in the nasal epithelium [12]. In the olfactory epithelium (OE), sustentacular cells exhibit high levels of ACE-2 expression [13], contrasting olfactory receptor neurons [14]. In brief, after successful infiltration, SARS-CoV-2 has the potential to restrict the innate immune response by dampening the interferon response in epithelial cells [15,16,17]. This leads to viral-induced inflammation of the olfactory mucosa, where circulating neutrophils and macrophages (CD68^+^ cells) infiltrate the infected tissue, resulting in an increase in cytokines [13,18,19,20]. However, it remains unclear whether the precise role of these cytokines is contributing to wound healing or engaging in tissue damage [20,21]. Additionally, when a successful immune response failed, a post mortem analysis of deceased patients with COVID-19 provided insights into immune response mechanisms. A 2022 cohort study revealed microvascular pathology and axon involvement in the olfactory mucosa of deceased patients [22]. However, few publications are available in post mortem studies regarding the immune response in the olfactory mucosa. Given the limited number of post mortem studies on the immune response in the olfactory mucosa, the present study aimed to address this gap by investigating the immune response through an immunohistochemistry analysis of tissue samples from the upper nasal turbinates of deceased patients with COVID-19 spanning from 2020 to 2022.

## 2. Results

### 2.1. Olfactory Mucosa and Neural Tissue

In order to analyze the immune response to COVID-19, the systemic immune responses of patients were examined before death. Therefore, the results of the blood analyses during their hospital stays were retrospectively collected (Table 1). As not all data could be retrieved, only one patient was included for the COVID-19^−^ group. Thus, no statistical analysis was performed. When compared to the reference blood levels (retrieved from the Carl Gustav Carus University Clinic of the TU Dresden), the COVID-19^+^ group showed increased mean levels of neutrophilic granulocytes with 24.14 GPt/L and C-reactive protein with 57.81 mg/L. The C-reactive protein levels showed an acute inflammatory response, which ultimately led to multiple lethal symptoms such as septic multi-organ failure, COVID-19 pneumonia, hypoxia, septic cardiovascular failure, and intracerebral hemorrhage, among others (see Table S1). Notably, the mean concentrations of lymphocytes were decreased, showing a value of 10.57% in the blood count.

**Figure 1:**
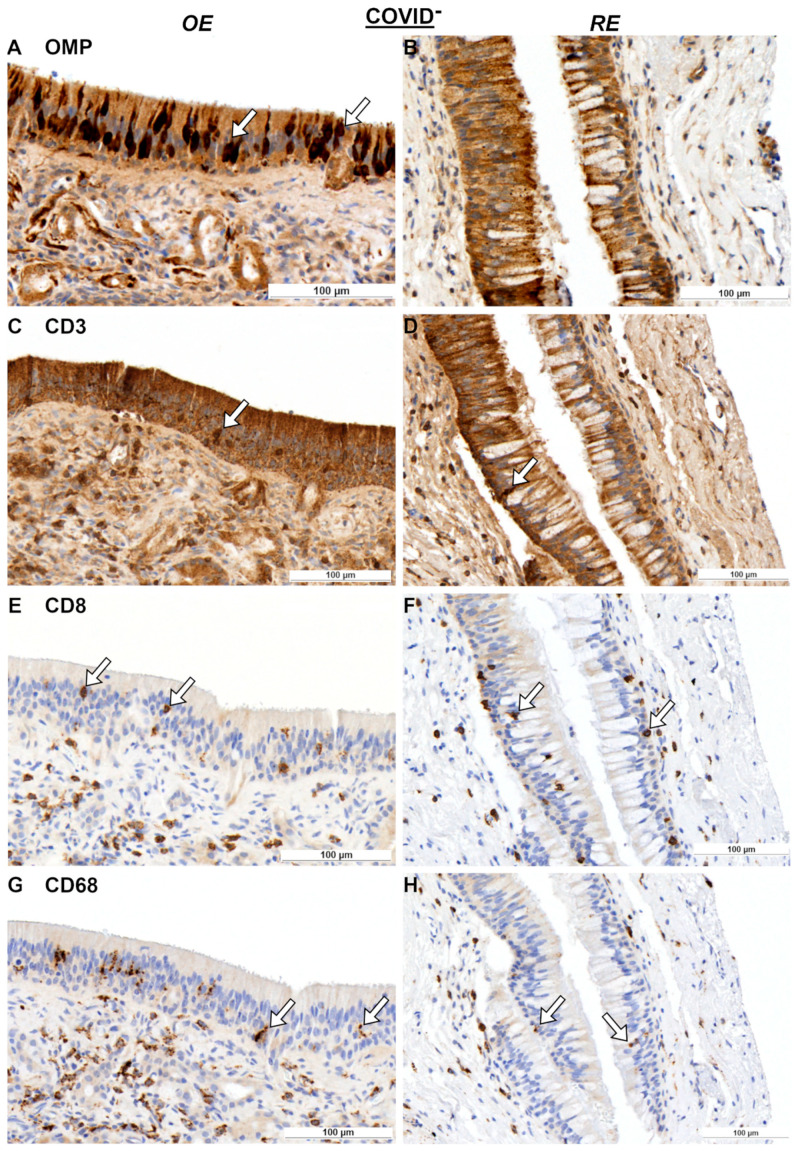
Immunohistochemical reactions in OE and RE for COVID-19^−^ specimens. OMP reactions for OE (**A**) and RE (**B**); CD3 reactions for OE (**C**) and RE (**D**); CD8 reactions for OE (**E**) and RE (**F**); and CD68 reactions for OE (**G**) and RE (**H**). Arrows indicate positive cells. Statistical evaluation of cell counting is shown in Figure 2. Scale bar is 100 µm. Negative controls are provided in Figure S1. Abbreviations: COVID^−^, control group.

**Figure 2:**
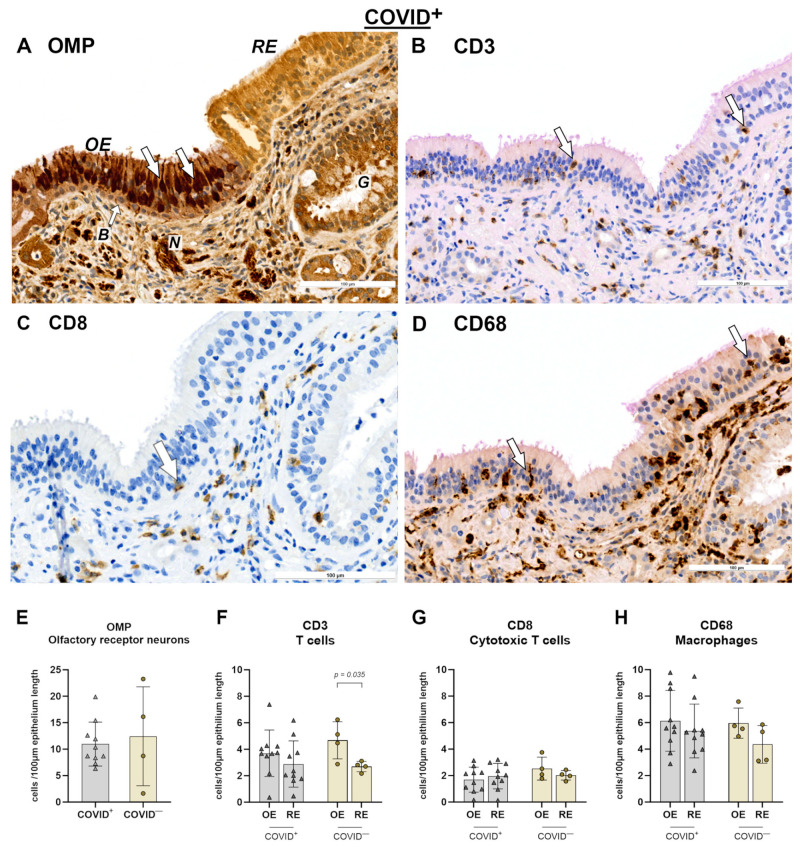
Immunohistochemical reactions in OE and RE for COVID-19^+^ specimens (**A**–**D**) and statistical evaluation of cell counts (**E**–**H**). (**A**) OMP reaction. Olfactory receptor neurons are indicated by arrows and form the olfactory epithelium (OE, epithelium is slightly red) [23]. Respiratory epithelium (RE) is slightly orange and does not show olfactory neurons. (**B**) CD3 reaction, arrows point at CD3- positive intraepithelial cells. (**C**) CD8 reaction (arrow). (**D**) CD68 reaction (arrows within the epithelium). For statistical analysis, cell counts of COVID-19^+^ and COVID-19^−^ specimens were compared using general linear model followed by multiple *t*-tests. Level of significance was set at α < 0.05. COVID-19^+^: *n* = 10. COVID-19^−^: *n* = 4. Scale bar is 100 µm. Negative controls are provided in Figure S2. Abbreviations: COVID^+^, patients with SARS-CoV-2 infection; COVID^−^, control group; B, basal cells of olfactory epithelium; B, basal cells; N, olfactory nerve; G, glands.

IHC was performed on nasal mucosa specimens in order to examine the local immunological alterations. Representative immunohistochemical images of the OE and respiratory epithelium (RE) are shown for olfactory marker protein (OMP), CD3, CD8, and CD68 in Figure 1A–H for the COVID-19^−^ group and in Figure 2A–D for the COVID-19^+^ group. First, OMP-positive cells were used to identify the OE and RE. For instance, Figure 2A shows the transition between both epithelia. Then, both the respiratory and olfactory epithelium lengths were measured for each specimen. The measured epithelium lengths of the COVID-19^+^ and COVID-19^−^ specimens did not significantly differ among the groups, assuming comparability (Figure 2E). Next, CD3, CD8, and CD68 positive cells were identified in both the OE and RE and were calculated as positive cells per 100 µm of epithelium length (Figure 2F–H). Notably, T cells (CD3^+^ cells) appeared to be more abundant in the lamina propria than in the epithelium. When comparing the OE to RE, a general linear model was calculated, which showed an effect based on the epithelial site. This effect was visible for T cells with *F*_6.20,1_ = 0.048 (Figure 2F) and for macrophages (CD68) with *F*_4.83,1_ = 0.048 (Figure 2H). Post hoc tests revealed that the number of T cells was higher in the OE when compared to that in the RE with *p* = 0.035 in the COVID-19^−^ group. Interestingly, this finding of higher T cells in the OE was absent for the COVID-19^+^ group. Also, the numbers of cytotoxic T cells (CD8^+^ cells) did not differ across the OE and RE (Figure 2G).

**Figure 3:**
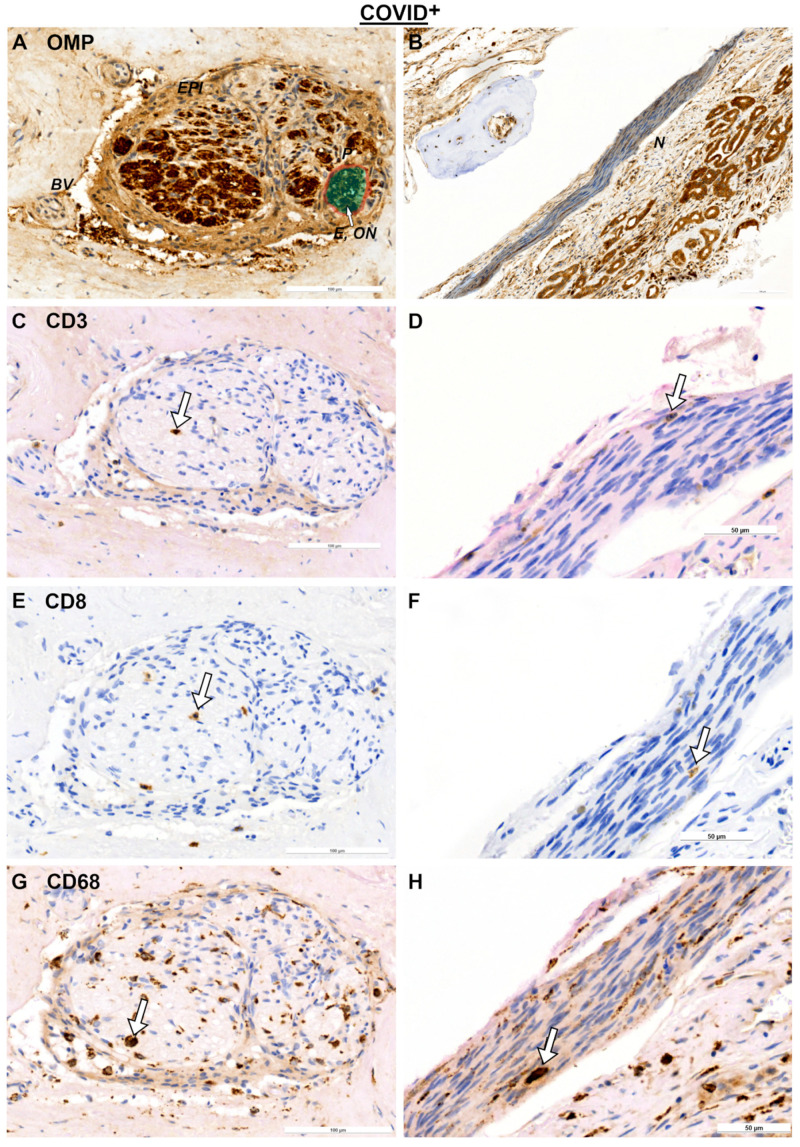
Immunohistochemical reactions in nerve tissue for COVID-19^+^ specimens. OMP reactions for ON (**A**) and N (**B**); CD3 reactions for ON (**C**) and N (**D**); CD8 reactions for ON (**E**) and N (**F**); and CD68 reactions for ON (**G**) and N (**H**). Arrows indicate positive cells. Statistical evaluation of cell counts is shown in Figure 4. Scale bars are 100 µm and 50 µm. Negative controls are provided in Figure S3. Abbreviations: E, endoneurium; EPI, epineurium, BV, blood vessel; COVID^+^, patients with SARS-CoV-2 infection; ON, olfactory nerve; N, trigeminal nerve.

**Figure 4:**
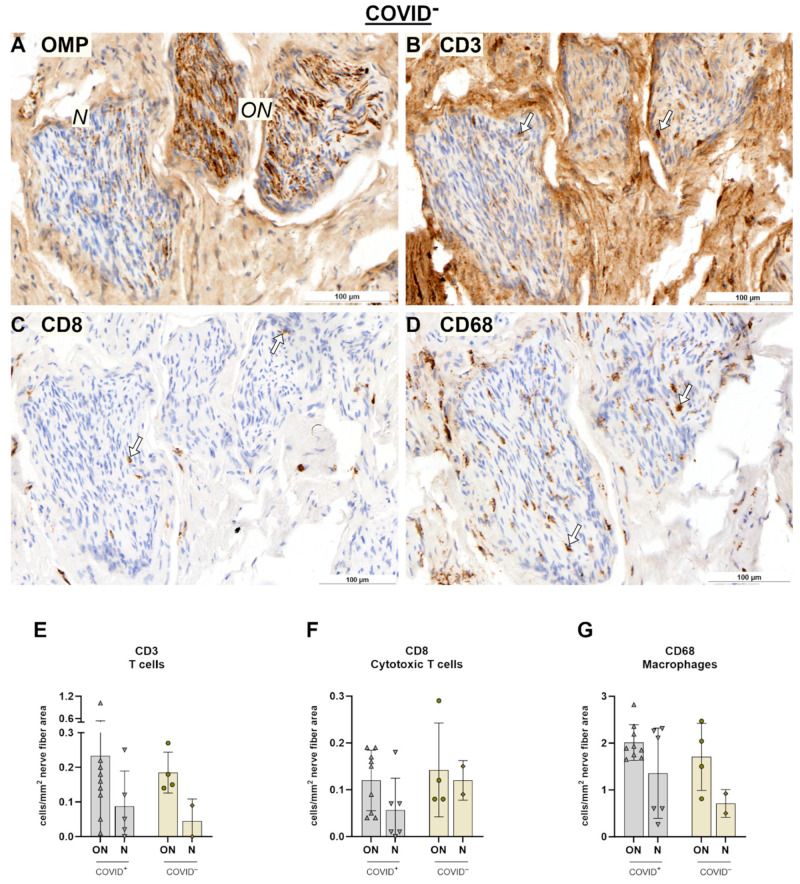
Immunohistochemical reactions in nerve tissue for COVID-19^−^ specimens (**A**–**D**) and statistical evaluation of cell counts (**E**–**G**). (**A**) OMP reaction. (**B**) CD3 reaction. (**C**) CD8 reaction. (**D**) CD68 reaction. Arrows indicate positive cells. For statistical analysis, cell counts of COVID-19^+^ and COVID-19^−^ specimens were compared using general linear model followed by multiple *t*-tests. Level of significance was set at α < 0.05. COVID-19^+^: ON, *n* = 9; N, *n* = 5. COVID-19^−^: ON, *n* = 4; N, *n* = 2. Scale bar is 100 µm. Negative controls are provided in Figure S4. Abbreviations: COVID^+^, patients with SARS-CoV-2 infection; COVID^−^, control group, ON, olfactory nerve; N, trigeminal nerve.

Concerning immunological responses after COVID-19 in other tissues, nerves were examined (Figure 3A–H and Figure 4A–D). For this purpose, nerves were classified as OMP^+^ nerves (ON) and OMP^−^ nerves, which are most likely trigeminal nerves (Figure 3A,B and Figure 4A). Descriptively, in transverse sections, CD3- and CD68- positive cells were found all over the endoneurium (Figure 3C,G), whereas CD8- positive cells were more restricted to the perineurium surrounding the endoneurium (Figure 3E). A general linear model showed an effect based on the nerve tissues with *F*_9.44,1_ = 0.037 for T cells (Figure 4E). T cells in the ON tended to be more abundant when compared to trigeminal nerves (*p* = 0.055). This trend was absent for cytotoxic T cells and macrophages (Figure 4F,G). Notably, there were only two samples showing trigeminal fibers, restricting further statistical analysis. In addition, the numbers of immune cells were sparse in the nerve fibers when compared to the epithelium, e.g., the mean values of CD3 positive cells were 0.22 ± 0.25 cells/mm^2^ for ON and 0.08 ± 0.09 cells/mm^2^ for trigeminal nerves. Therefore, it appeared that the immune response in the nerve fibers was not pronounced.

### 2.2. Olfactory Bulb (OB) and SARS Spike Protein

**Figure 5:**
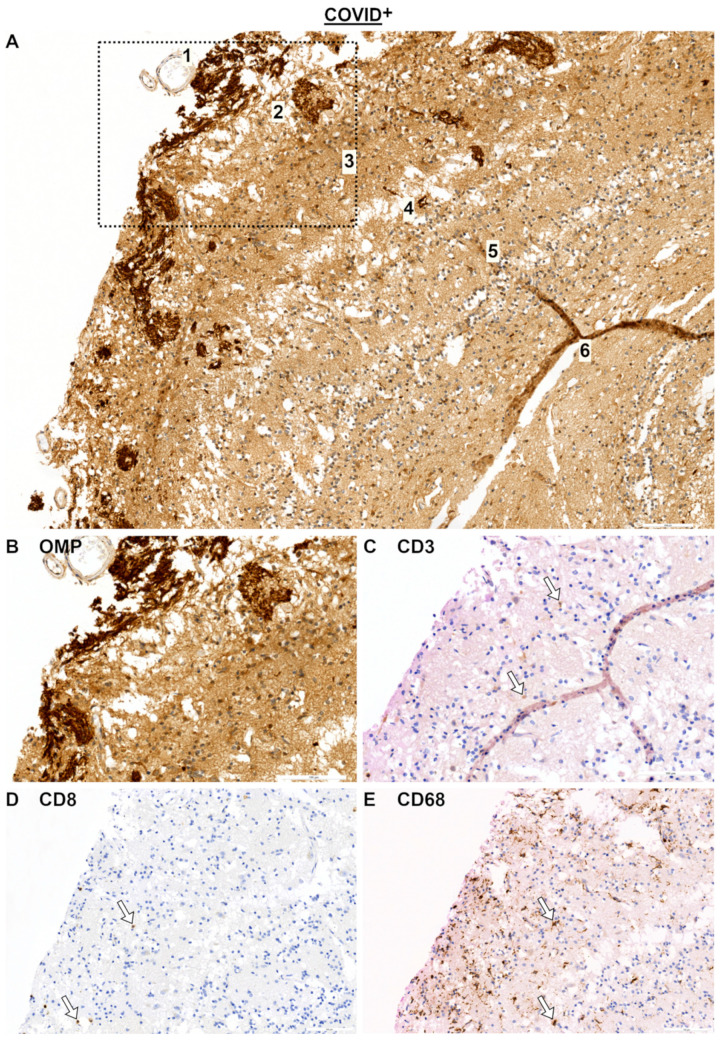
Immunohistochemical reactions in OB for COVID-19^+^ specimens (**A**,**B**) OMP reaction. Numbers indicate OB layers: 1, nerve fiber layer (not fully pictured); 2, glomerular layer; 3, external plexiform layer; 4, mitral/tufted cell layer; 5, internal plexiform layer; 6, granule cell layer [24]. (**C**) CD3 reaction. (**D**) CD8 reaction. (**E**) CD68 reaction. Arrows indicate positive cells. Scale bar: 100 µm. Negative controls are provided in Figure S5. Abbreviations: COVID^+^, patients with SARS-CoV-2 infection.

In one specimen, the OB was found showing typical stratification (Figure 5A). Descriptively, when localizing the CD3, CD8, and CD38 immune cell responses, positive cells were found in the glomerular and external plexiform layers. The macrophages exhibited a dense pattern in the glomerular layer.

**Figure 6:**
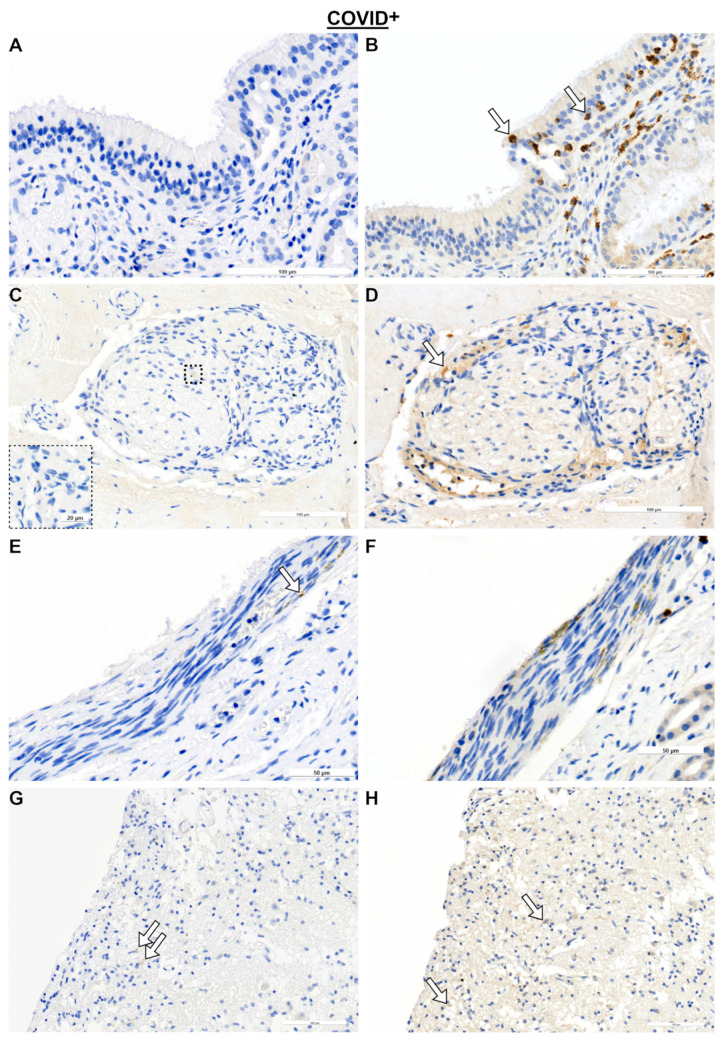
Immunohistochemical reactions in OE and RE, nerves, and OB for COVID-19^+^ specimens. SARS spike protein reactions in epithelium (**A**), nerve fiber bundles (**C**,**E**), and OB (**G**). ACE-2 reactions in epithelium (**B**), nerves (**D**,**F**), and OB (**H**). Arrows indicate positive cells. Negative controls are provided in Figure S7. Abbreviations: COVID^+^, patients with SARS-CoV-2 infection.

SARS spike protein and ACE-2 were also investigated descriptively in the OE and RE, ON, trigeminal nerves, and OB. Representative pictures are shown in Figure 6. Although SARS spike protein was not detected in the epithelium in this specimen (Figure 6A), other specimens showed positive reactions for SARS spike protein (Figure S6). The nerve tissues showed reactions for SARS spike protein, which was more restricted to the perineurium (Figure 6C,E). Specifically, the trigeminal nerves revealed SARS spike protein. Interestingly, SARS spike protein was not detectable in the lamina propria, nor was it adjacent to blood vessels. The OB also showed positive signs of SARS spike protein in the glomerular layer (Figure 6G). In addition, SARS spike protein was observed in arterial and venous blood.

ACE-2 was detected in the RE and in the epineurium of ON cells (Figure 6B,D). Also, ACE-2 was frequently visible in blood vessels. The OB showed a more dispersed pattern of positive ACE-2 cells, most likely adjacent to arterioles [23].

## 3. Discussion

The present study aimed to investigate the immune response in the nasal mucosa and OB of deceased patients with COVID-19 via immunohistochemistry (IHC). There were several key observations. Firstly, patients who succumbed to severe COVID-19 infection displayed a local decrease in T lymphocytes within the OE. Secondly, nerve fibers, both olfactory and trigeminal, exhibited an immune response. This immune response was also visible in the glomerular layer of the OB. Thirdly, signs of SARS spike protein were detectable in olfactory and trigeminal nerve fibers, as well as in the OB, but they were almost absent in the epithelium. The results showed peripheral lymphopenia in severe COVID-19 cases, with a localized reduction in T cells (CD3^+^ cells) in the OE compared to deceased patients without COVID-19. It is essential to note that our study combined data from deceased patients between 2020 and 2022, involving SARS-CoV-2 before the WHO classified variants of concern and five different variants of concern of SARS-CoV-2 [25]. Symptom prevalence varies depending on the virus strain, as demonstrated by Whitaker and colleagues [26], who predicted a distinct symptom profile for the Omicron variant compared to the Alpha variant in a study on the English population from 2020 to 2022.

### 3.1. Immune Response in Nasal Mucosa

Concerning peripheral lymphopenia in severe COVID-19 cases, Candia et al. [27] reported that lower CD4 and CD8 cell blood counts are associated with a higher ratio of severe COVID-19 cases [28,29]. Additionally, a 2023 study utilizing a spherometer [30] to compare T cell responses in mRNA-vaccinated participants to patients with COVID-19 revealed a decrease in blood cell counts of T lymphocytes [31]. These findings align with the present results, showing a decreased number of T lymphocytes. As the pandemic progressed, studies from 2021 to 2022 indicated that infection or vaccination status significantly protected against severe disease but not against re-infection [32,33,34]. Notably, in a Syrian hamster model, re-infection after vaccination resulted in the presence of SARS-CoV-2 in the upper nasal airways, indicating a lack of successful protection [35]. This led to the proposition of potential incomplete protection in the upper nasal airways [21]. Indeed, Wellford et al. [36] proposed the existence of a tight endothelial structure forming a blood–olfactory barrier that segregates the olfactory from the respiratory mucosa. In their study, they intraperitoneally injected mice with antibodies targeting both epithelia, revealing immunohistochemical reactions exclusively in RE cells [36]. In this context, a decrease in total T cells (CD3^+^ cells) was observed in the OE in COVID-19 cases when compared to deceased non-COVID-19 controls, while cytotoxic T cells remained unchanged.

Currently, there are few studies about the post mortem COVID-19-induced T cell response in the olfactory mucosa (e.g., Kirschenbaum et al. [37]), especially with respect to the comparison between such cases and a non-COVID-19 control group. For instance, Finlay et al. reported an enrichment of resident cytotoxic T cells in biopsies from patients suffering from long-term COVID-19 with olfactory dysfunction [38]. Comparing the results from this study to the present investigation is problematic, as the present investigation focuses on severe COVID-19 cases with fatal outcomes. Moreover, regretfully, the present study lacks data on both the qualitative and quantitative aspects of olfactory function during patients’ hospitalization. Consequently, drawing conclusions from these distinct study cohorts is challenging. In another study, Roukens et al. [39] investigated immune cells in swabs from the nasal inferior turbinate via flow cytometry. During acute COVID-19 infection, the numbers of T effector and natural killer cells increased, whereas two months after infection, the number of SARS-CoV-2-specific cytotoxic T cells was elevated [39]. The results from the present study align with this finding because altered cytotoxic T cells were not observed during acute COVID-19 infection. Apart from that, there was only one study which reported the presence of cytotoxic T cells only in patients with milder COVID-19 infections [30].

### 3.2. Immune Responses in Nerve Fibers and Central Nervous System

When profiling the immune responses in the nasal mucosa and central nervous system, different studies identified an increase in early macrophages in the OE [40] of T_H_ (CD4^+^) cells [41] and of cytotoxic T cells in the brain stem, parenchyma, and different brain regions in response to COVID-19 [40,42,43]. Descriptively, the present study suggests an infiltration of T cells and macrophages in the glomerular layer of the OB and a trend of an increased T cell response in the olfactory nerves when compared to trigeminal nerves. This finding is accompanied by positive SARS spike protein reactions in nerve fibers and the OB. In line with the current observations, Meinhardt et al. showed the presence of SARS-CoV-2 in the olfactory mucosa, in olfactory and trigeminal nerve fibers, and in some samples in the central nervous system [40]. This, in turn, supports the neuroinvasive potential of SARS-CoV-2, leading to inflammatory olfactory neuropathy in some cases [44]. While neuroinvasion is a documented aspect of the COVID-19 pathology, the likelihood of infection from the olfactory route to the CNS via olfactory fibers is considered low [14]. Khan et al. showed that sustentacular cells are infected by SARS-CoV-2, leading to degradation; they did not report a direct infection of the olfactory sensory neurons themselves [14,45,46]. In addition, contrary to the present study, no SARS-CoV-2 signs were detected in the OB [14]. In a recently published review by Meinhardt et al. (2024), the authors supported the likelihood of a SARS-CoV-2-induced leakage of the blood–brain barrier [45]. Signs of blood–brain barrier leakage and endothelial inflammation were found in patients dying with or from COVID-19, and an upregulation of T cells was found in the perivascular space adjacent to endothelial cells [22,47,48,49,50]. The neuroinvasive potential of COVID-19 is clinically indicated by neurological symptoms such as headache, brain fog, and a change in or loss of smell (anosmia) [26,51]. In the case of anosmia, the olfactory nerve remained largely intact, revealing other factors as the cause of anosmia, e.g., the degradation of sustentacular cells supporting olfactory receptor neurons or the obstruction of the olfactory cleft [52]. Moreover, with regard to olfactory dysfunction, little is reported about trigeminal sensitivity [53]. One study in 2022 reported a correlation between olfactory dysfunction and reduced nasal chemesthesis [54]. The reduction in chemesthesis—a sensation of nasal obstruction—is related to trigeminal function [55]. However, due to the limited sample size in the present study, it appears to be problematic to draw more conclusions about affections of the nasal trigeminal system.

In summary, the present post mortem investigation revealed a decreased T cell response in patients with COVID-19 and signs of SARS-CoV-2 presence in olfactory and trigeminal fibers.

### 3.3. Limitations

Krasemann et al. published guidelines on how to investigate the presence of SARS-CoV-2 in human autopsy tissues [48]. As a gold standard, the authors suggested a screening with quantitative PCR to detect tissues with high viral contents, followed by IHC and in situ hybridization. At the end, to verify the presence of the virus and not only of the virus capsid or SARS spike protein, electron microscopy should be performed. This workflow revealed 135 misinterpretations out of 144 cases [48]. Due to preservation reasons, the suggested methods could not be performed. On the other hand, the present results confirm previously published findings using the more accessible method of IHC. In addition, the used SAR-CoV-2 antibody appears to show a low signal. In the study by Krasemann et al., this antibody did not lead to any reactions in the tissue. However, the authors reported that this frequently used antibody showed reactions in other studies [48]. Another limitation of this study is the low sample size. Although the initial sample size was *n* = 65, most tissue samples showed no OE. This finding is most likely related to the age-dependent metaplasia of the epithelium and degeneration processes in older humans [56].

## 4. Materials and Methods

### 4.1. Study Design and Group

This study was approved by the Ethics Committee at the Carl Gustav Carus University Clinic of the TU Dresden (application number: BO-EK-175052020) and conducted following the principles for medical research involving human subjects as described in the Declaration of Helsinki [57]. To investigate the effect of SARS-CoV-2 on cellular processes, samples from the olfactory cleft and/or upper turbinate were retrieved from deceased patients from the Institute of Pathology at the TU Dresden from November 2020 to March 2022. During this period, the following SARS-CoV-2 variants were recorded in Germany: Alpha (B.1.1.7) from January to September 2021, Beta (B.1.351) from January to July 2021, Gamma (B.1.1.28) from February to July 2021, Delta (B.1.617.2) from April 2021 to March 2022, and Omikron (B.1.1.529) since November 2021 [58,59].

**Figure 7:**
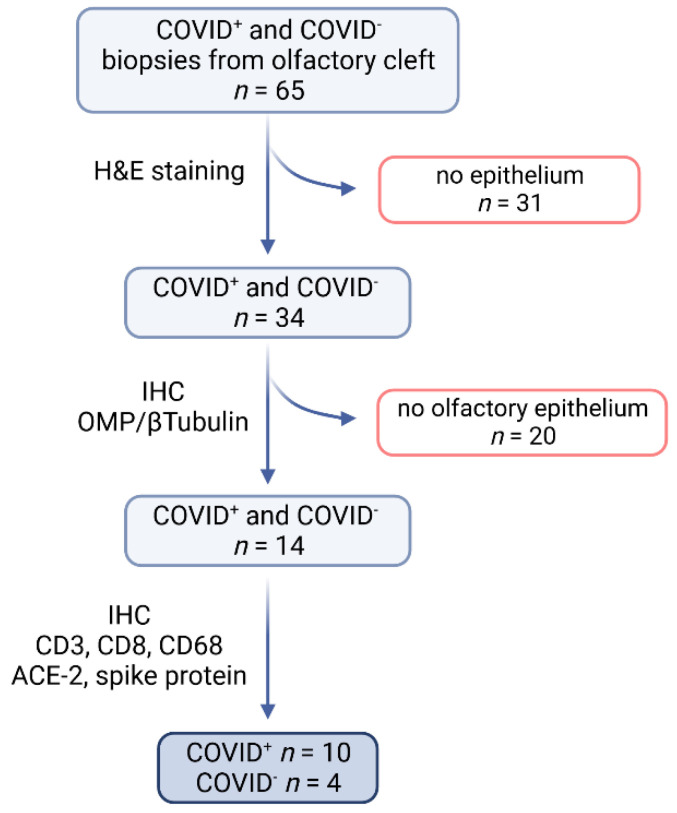
The pipeline of sample processing. A total of 65 specimens were taken from patients with (*n* = 42) and without (*n* = 23) COVID-19 at the time of death. The specimens were screened via H&E staining for epithelium. A total of 31 specimens showed no epithelium. Then, in order to detect olfactory epithelia, the specimens were visualized immunohistochemically with OMP and β-Tubulin antibodies, resulting in 20 dropouts. At the end, *n* = 10 specimens had a confirmed infection of COVID-19 (COVID-19^+^), and *n* = 4 specimens were used as controls without COVID-19 infection (COVID-19^−^) and were analyzed for immunological markers (CD3, CD8, and CD68) as well as for SARS-CoV-2 spike protein and ACE-2.

In order to sample the olfactory cleft and/or upper turbinate, a craniectomy was performed. Scalp skin was removed to expose the skull, which was then opened with a bone saw. The calvaria and brain were removed. Following this, the OB was removed together with the cribriform plate. In two cases, the OB was incidentally preserved and subjected to a qualitative analysis. At the end, a tissue specimen was taken from the area of the olfactory cleft and/or upper turbinate and stored directly in 4 % formalin for further investigation. In total, 65 specimens were taken from patients with SARS-CoV-2 infection (*n* = 42; referred to as COVID-19^+^) and without COVID-19 symptoms (*n* = 23; control group, referred to as COVID-19^−^). At this stage, the evidence of SARS-CoV-2 was tested in the clinic via a PCR test. Then, all tissue specimens were screened for epithelia via routine H&E staining (Merck, Darmstadt, Germany and Carl Roth, Karlsruhe, Germany). In order to detect olfactory epithelia (OE), the remaining 34 specimens were immunohistochemically visualized with olfactory marker protein (OMP) and β-tubulin antibodies. This yielded a total dropout of 51 specimens. In order to verify an acute COVID-19 infection, all specimens were subjected to IHC for the spike protein of SARS-CoV-2, resulting in *n* = 10 for the COVID-19^+^ samples and *n* = 4 for the COVID-19^−^ samples. Finally, all 14 specimens reacted with immunological markers (CD3, CD8, and CD68), and the COVID-19^+^ specimens were additionally analyzed for the receptor ACE-2. Notably, four of the patients who tested negative for SARS-CoV-2 by PCR upon admission to the clinic were found to be positive for SARS spike glycoprotein reactions. Therefore, we divided the groups based on the immunohistochemical reactions (Figure 7). A description of the study group is provided in Table 1. A further description of the pathological background of the cohort is provided in Table S1.

### 4.2. Sample Processing and Selection

The specimens were fixed overnight in 4 % formalin, decalcified with Osteosoft (Merck, Darmstadt, Germany) for 24–72 h, dehydrated, and embedded in paraffin.

For preservation of OE, paraffin blocks were cut into 30 sections with a 5 μm thickness (Leica Jung RM 2065, Wetzlar, Germany), mounted on slides (EPREDIA Superfrost Plus Adhesion, Basel, Switzerland), and H&E stained at 50 μm intervals. If OE was detected, the adjacent slides were also processed. Immunohistochemical reactions were performed for CD3, CD8, CD68, SARS spike glycoprotein, and ACE-2, along with their respective negative controls. Subsequently, the specimens were screened for nerve fibers. Nerves that tested positive for OMP were classified as olfactory fibers (ON), while those that only tested positive for ß-tubulin were classified as trigeminal nerves (N). It is important to note that the latter may also be aged by degenerating ON.

### 4.3. Immunohistochemistry (IHC)

The immunohistochemical procedures are described in detail in Protocol S9. Briefly, specimens were deparaffinized in xylene and hydrated. Then, antigen retrieval was performed with a microwave step and the corresponding buffer, as listed in Table 2. Afterwards, endogenous peroxidases were inhibited with 3 % H_2_O_2_ (Merck, Darmstadt, Germany) followed by the blocking of non-specific binding sites with 5 % normal goat serum (Vector Laboratories, Newark, CA, USA). According to Table 2, primary antibodies were diluted and incubated overnight at 4 °C. Subsequently, specimens were incubated with secondary antibodies (see Table 2) and ABC-HRP kit (Vectastain Elite, Newark, CA, USA). Visualization was performed by adding 3,3′-diaminobenzidine–tetrahydrochloride (DAB; Sigma Aldrich, Merck, Darmstadt, Germany) for 7 min. After stopping the reaction in tap water, counterstaining with hematoxylin was performed for less than 10 s. Specimens were then dehydrated, mounted in DePeX (Serva, Heidelberg, Germany), and coverslipped. Negative controls were performed for each reaction while omitting the primary antibody.

### 4.4. Quantification and Statistical Analysis

Slides were scanned with a wild field slide scanner microscope (Zeiss Axio Scan.Z1, Carl Zeiss Microscopy GmbH, Jena, Germany) equipped with an HV-F202SC color CCD camera (dexel size of 4.4 µm; Hitachi Kokusai Electric Europe GmbH, Frankfurt, Germany). Single pictures used as negative controls were recorded with a Zeiss Scope A1 microscope (Carl Zeiss Microscopy GmbH, Jena, Germany) equipped with an Axiocam 503 color camera (Carl Zeiss Microscopy GmbH, Jena, Germany). For recording, Zeiss ZEN 3.1 blue software was used. All slides and pictures were recorded at 20× magnification.

Epithelia and nerve tissues were analyzed with QuPath 0.4.4 (Copyright 2018–2022 QuPath developers, the University of Edinburgh). First, for epithelium measurements, the whole slide was screened for OMP-positive reactions in the OE. Then, equivalent OE sites were identified for CD3, CD8, and CD68 reactions. This procedure was followed for respiratory epithelium (RE). In total, a mean of 4.86 ± 2.49 sites for OE and 4.00 ± 1.13 sites for RE per slide were quantified. Positive reacting cells per 100 µm epithelium length were counted. Nerve fibers were processed as described above, while immunological responses were compared between ON and N. Means of 24.17 ± 15.47 ON structures and 4.59 ± 7.35 N structures were analyzed. To compare ON and N, the nerve area was measured, and positive cells per 1 mm^2^ nerve area were determined. Immunohistochemical reactions with ACE-2 and SARS spike glycoprotein antibodies were not quantifiable. Therefore, the reactions in the epithelium, nerve tissue, and OB are presented descriptively.

All images were processed in GIMP (version 2.10.36, GNU Image Manipulation Program, https://www.gimp.org/, accessed on 18 January 2023), and coloring of epithelia was performed in Adobe Photoshop (version 23.1.0, Adobe Systems Software Ireland Limited, Dublin, Ireland).

Statistical analysis was performed with IBM SPSS Statistics (version 28.0.1.0; IBM, Chicago, IL, USA). First, group homogeneity was verified for age and OE length among groups. Pearson’s Chi square test showed no significant differences. One-way ANOVA showed a significant difference in the group size. Due to the small sample size for the COVID-19^−^ group, Shapiro–Wilk test for normal distribution comparison could not be applied. In order to compare an effect of the positive stained cells per 100 µm epithelium or per mm^2^, general linear model was performed. Then, due to the small sample size, multiple unpaired *t*-tests were performed. Level of significance was set at α < 0.05. All data were visualized with Graphpad Prism (version 10.1.2 (324); Boston, MA, USA), and the data are shown as mean ± standard deviation (SD) if not stated differently.

## Figures and Tables

**Table 1:** Descriptive statistics of study groups.

		COVID^+^					Reference Values	COVID^−^				
		*n*	*Mean*	*SD*	*Max*	*Min*		*n*	*Mean*	*SD*	*Max*	*Min*
**Gender**	*female*	3						3				
*male*		7						1				
**Age [years]**		10	78.5	12.55	94	58		4	73.5	12.66	85	56
**Weight [kg]**		10	63.1	12.94	83	47		4	77.5	16.86	92	54
**Days between death and autopsy**		10	4.8	5.33	19	1		4	4.25	2.5	7	1
**Blood parameters**												
Leukocytes [GPt/L]		8	8.19	5.22	17.69	1.05	3.8–9.8	2	8.61	3.87	11.35	5.87
Neutrophilic granulocytes [%]		5	77.76	11.99	91.4	66.8	36.0–77.0	1	48.7	-	-	-
Neutrophilic granulocytes [GPt/L]		5	**24.12** **↑**	37.96	91.4	2.82	1.80–7.55	1	5.53	-	-	-
Lymphocytes [%]		6	**10.57** **↓**	4.24	15.4	5.3	20.0–49.0	1	43.7	-	-	-
Lymphocytes [GPt/L]		6	3.14	6.25	15.89	0.16	1.50–4.00	1	**4.96** **↑**	-	-	-
Monocytes [%]		6	8.83	8.09	21.8	1	0.0–9.0	1	6.3	-	-	-
Monocytes [GPt/L]		6	0.82	0.76	2.1	0.01	0.20–1.00	1	0.71	-	-	-
C-reactive protein [mg/L]		8	**57.81** **↑↑**	74.76	211	1.6	<5.0	3	3.33	1.72	5.3	2.1

Bold numbers indicate values out of reference values. **↓,↑** moderately deviating value; ↑↑, highly increased value. Abbreviations: *n*, number of patients; COVID-19^+^, confirmed infection with COVID-19; COVID-19^−^, control group.

**Table 2:** Antibody dilutions. Secondary antibodies were produced in goat and were diluted at a ratio of 1:200.

Primary Antibody	Company (No./Clone No.)	Dilution	Secondary Antibody	Company	Buffer
**OMP** **(rabbit)**	Sigma-Aldrich (Darmstadt, Germany) (MFCD09265364)	1:6000	Biotinylated gt-anti-rabbit	Vectorlabs (VEC-BA-1000)	TRIS-EDTA
**Beta-Tubulin** **(mouse)**	BioLegend (Amsterdam, Netherlands) (TUBB3; TUJ1))	1:1000	Biotinylated gt-anti-mouse	Vectorlabs (VEC-BA-9200)	TRIS-EDTA
**CD3** **(rat)**	abcam (Cambridge, UK) (CD3-12)	1:250	Biotinylated gt- anti-rat	Vectorlabs (VEC-BA-9400)	TRIS-EDTA
**CD8** **(mouse)**	abcam (C8/144B)	1:100	Biotinylated gt-anti-mouse	Vectorlabs (VEC-BA-9200)	TRIS-EDTA
**CD68** **(mouse)**	abcam (KP1)	1:500	Biotinylated gt-anti-mouse	Vectorlabs (VEC-BA-9200)	TRIS-EDTA
**ACE2** **(rabbit)**	abcam (Anti-ACE2 antibody, ab15348)	1:4000	Biotinylated gt-anti-Rb	Vectorlabs (VEC-BA-1000)	Citrate
**SARS spike glycoprotein (mouse)**	abcam (3A2)	1:100	Biotinylated gt-anti-mouse	Vectorlabs (VEC-BA-9200)	Citrate

## Data Availability

All raw data can be provided upon request from the corresponding author.

## Supplementary Materials

The following supporting information can be downloaded at https://www.mdpi.com/article/10.3390/ijms25084427/s1.
