# Supplementary material for: Histochemical Evidence for Reduced Immune Response in Nasal Mucosa of Patients with COVID-19
Source: Int J Mol Sci. 2024 Apr 17;25(8):4427. doi: 10.3390/ijms25084427 (PMC11050322; doi:10.3390/ijms25084427)
Supplement: Supplementary file 1 [file ijms-25-04427-s001.zip › ijms-2929541-supplementary.pdf]

Table S1: Clinical diagnosis and pathological results of the study group.

	COVID+<br>n = 10	COVID-<br>n = 4
**Clinical diagnosis#**		
general	SARS-CoV- 2 infection, Acute and chronic renal failure, purulent pyelonephritis, septic multi-organ failure, organic pneumonia	multiple organ failure, shock, haemmorhag. shock, infection
respiratory diagnosis	aspiration pneumonia, Respiratory insufficiency, atypical pneumonia, COVID-19 pneumonia, retention pneumonia, hypoxia,	respiratory insufficiency, pulmonary emphysema,
cardio-vascular diagnosis	septic cardiovascular failure, subacute myocardial infarction, acute global heart failure, critical myocardial hypertrophy, cardiorespiratory insufficiency	cardiac insufficiency, ruptured aneurysm dissecans (thoracic aorta), global hypoxemic cardiovascular failure, acute global heart failure, atherosclerosis of the aorta
neurological diagnosis	intracerebral haemorrhage, haemorrhagic cerebral infarction of basal ganglia, dementia	
others	undifferentiated non-small cell carcinoma of the right lung with focal neuroendocrine differentiation	covered perforated abdominal aortic aneurysm, Meningeosis leucaemica
**Pathological results#**		
neuropathological results	cerebral oedema, Morbus Alzheimer, neuritic plaques, accumulation Tau protein, neurofibrillary tangles, basal cerebral arteriosclerosis, hyaline angiopathy with encephalopathy, isolated eosinophilic, pyknotic nerve cells in the brain and cerebellum, subdural and parenchymal haemorrhage, haemorrhagic infarction of basal ganglia, perivascular isolated lymphocytic infiltrate	cerebral oedema, neuritic plaques, accumulation of Tau protein, neurofibrillary tangles, basal cerebral arteriosclerosis, haemorrhage
cardio-vascular results	arteriosclerosis with stenosis	arteriosclerosis
otolaryngological results	nasal sinus: low-grade chronic rhinosinusitis	

#summary of most common findings examined during autopsy

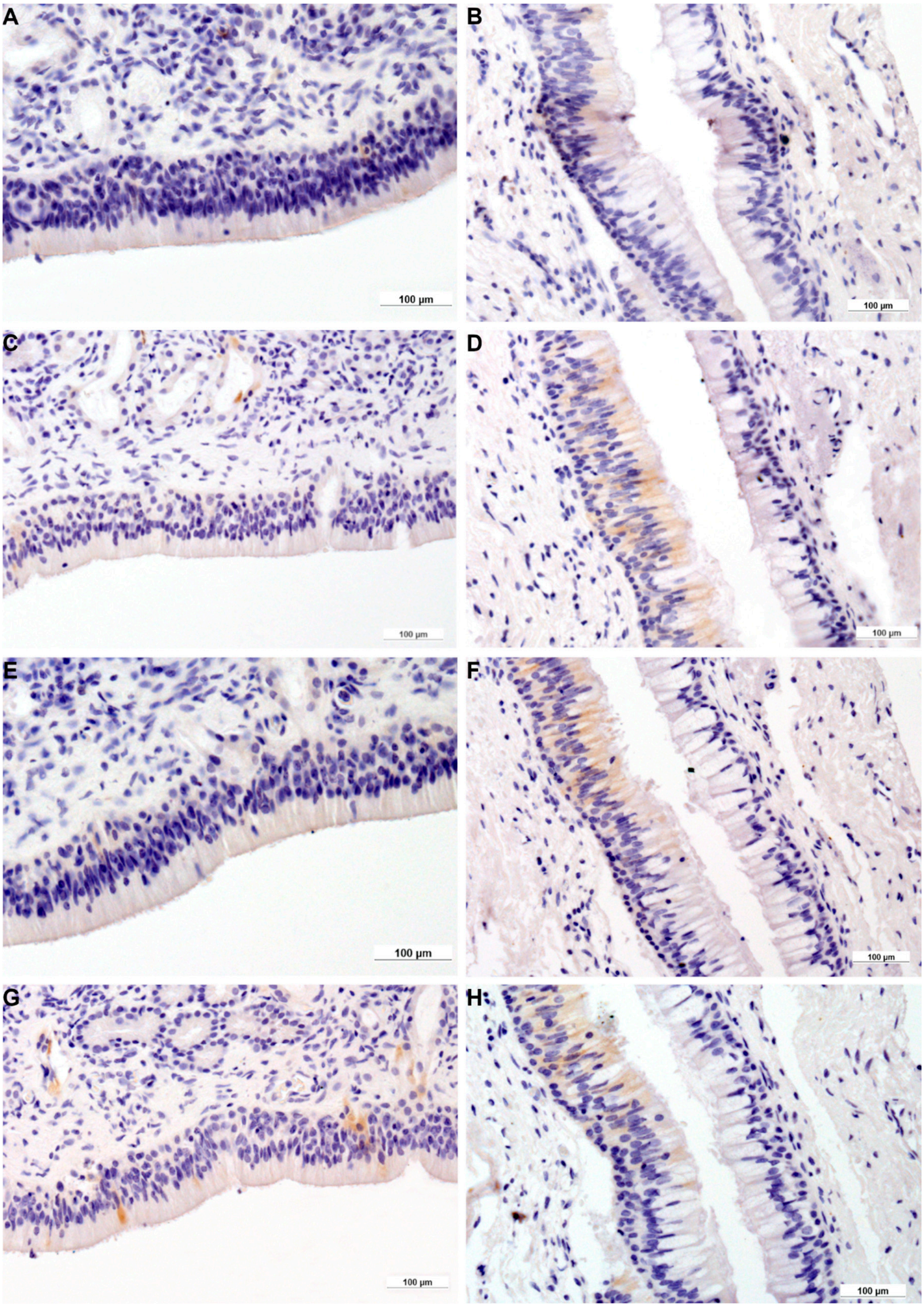

Figure S1: Negative controls for Figure 1, COVID⁻, immunohistochemical reactions in OE and RE. Controls for: OMP reactions for OE (**A**) and RE (**B**); CD3 reactions for OE (**C**) and RE (**D**); CD8 reactions for OE (**E**) and RE (**F**); and CD68 reactions for OE (**G**) and RE (**H**). Scale bar 100 µm. Abbreviations: COVID⁻, control group; OMP, olfactory marker protein; CD3, marker for T cells; CD8, marker for cytotoxic T cells; CD68, marker for macrophages.

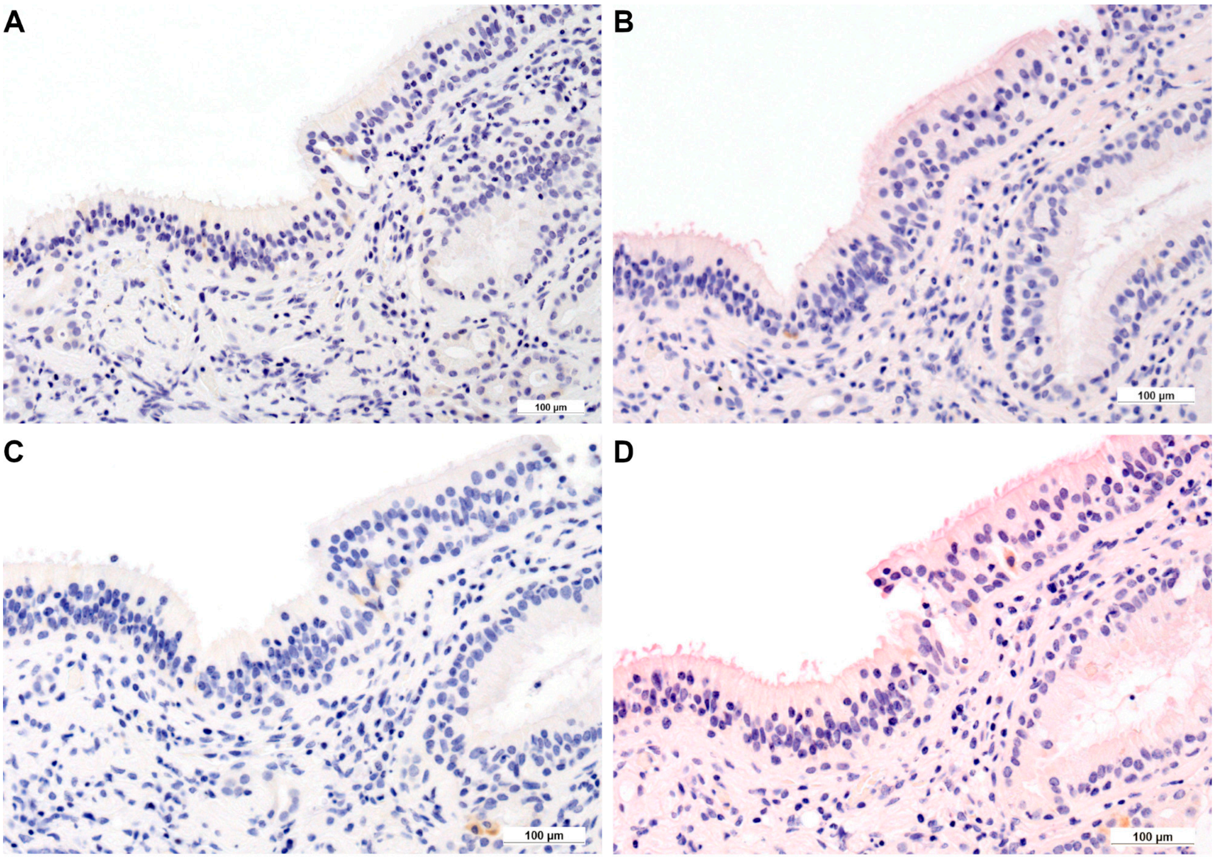

Figure S2: Negative controls for Figure 2, COVID⁺, immunohistochemical reactions in OE and RE. Controls for: OMP reaction (**A**), CD3 reaction (**B**) CD8 reaction (**C**), and CD68 reaction (**D**). Scale bar 100 µm. Abbreviations: COVID⁺, patients with SARS-CoV-2 infection; OMP, olfactory marker protein; CD3, marker for T cells; CD8, marker for cytotoxic T cells; CD68, marker for macrophages.

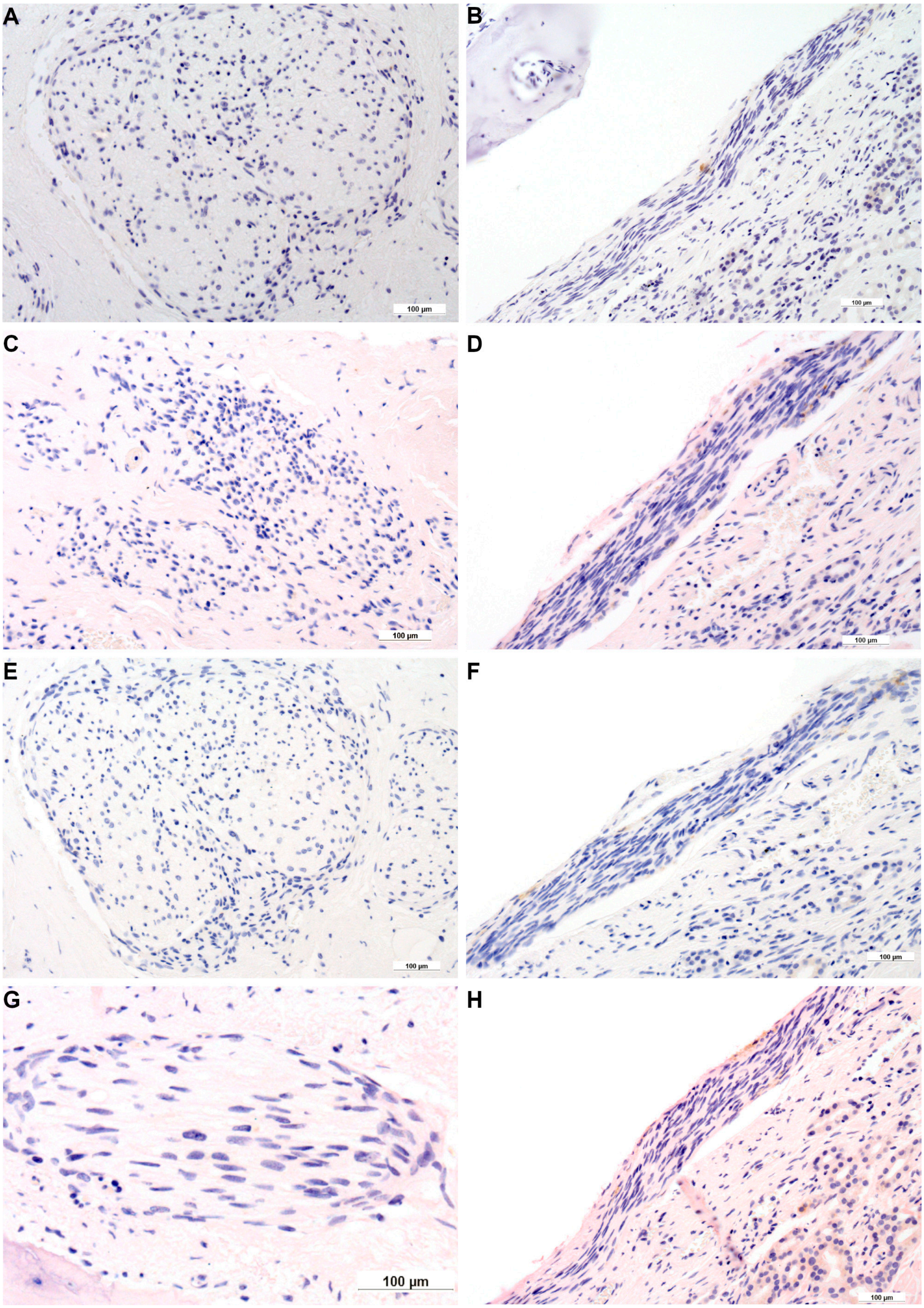

Figure S3: Negative controls for Figure 3, COVID$^+$, immunohistochemical reactions in nerve tissue. Controls for: OMP reaction (**A,B**), CD3 reaction (**C,D**), CD8 reaction (**E,F**), and CD68 reaction (**G,H**). Scale bar 100 µm. Abbreviations: COVID$^+$, patients with SARS-CoV-2 infection; OMP, olfactory marker protein; CD3, marker for T cells; CD8, marker for cytotoxic T cells; CD68, marker for macrophages.

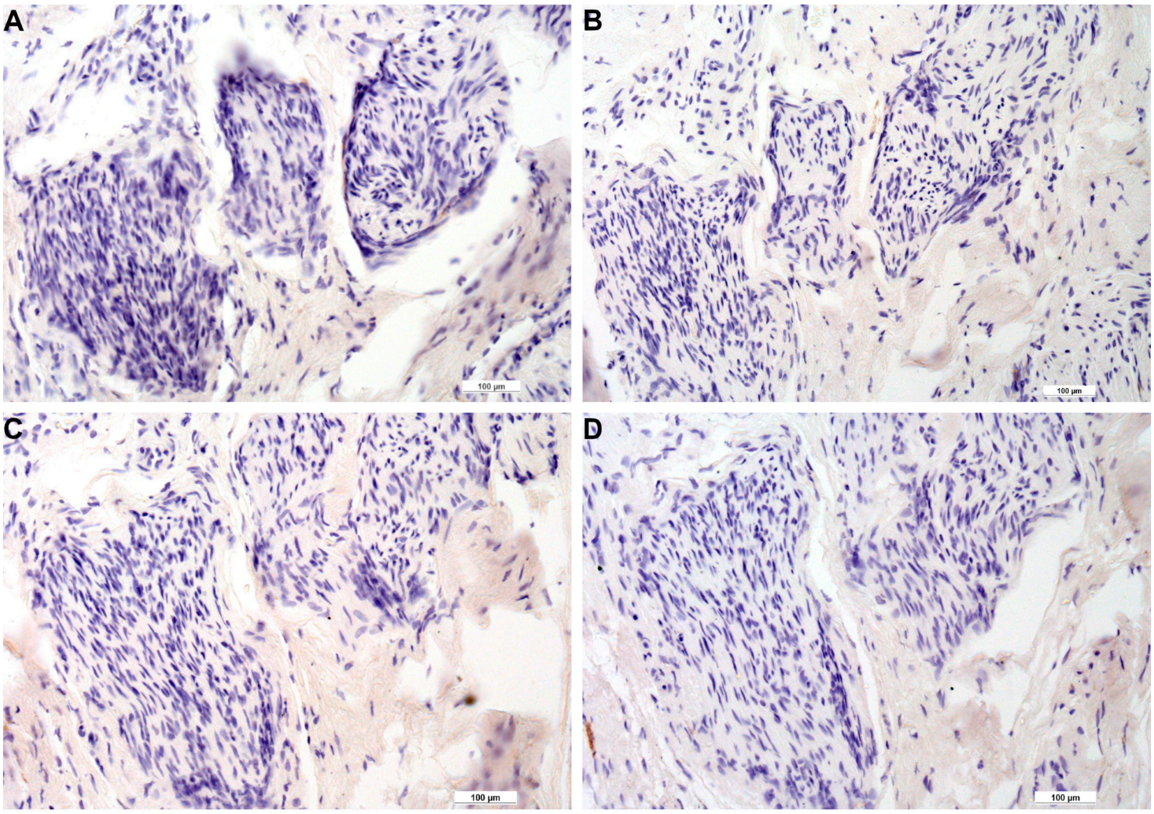

Figure S4: Negative controls for Figure 5, COVID⁻, immunohistochemical reactions in nerve tissue. Controls for: OMP reaction (**A**), CD3 reaction (**B**) CD8 reaction (**C**), and CD68 reaction (**D**). Scale bar 100 µm. Abbreviations: COVID⁻, control group; OMP, olfactory marker protein; CD3, marker for T cells; CD8, marker for cytotoxic T cells; CD68, marker for macrophages.

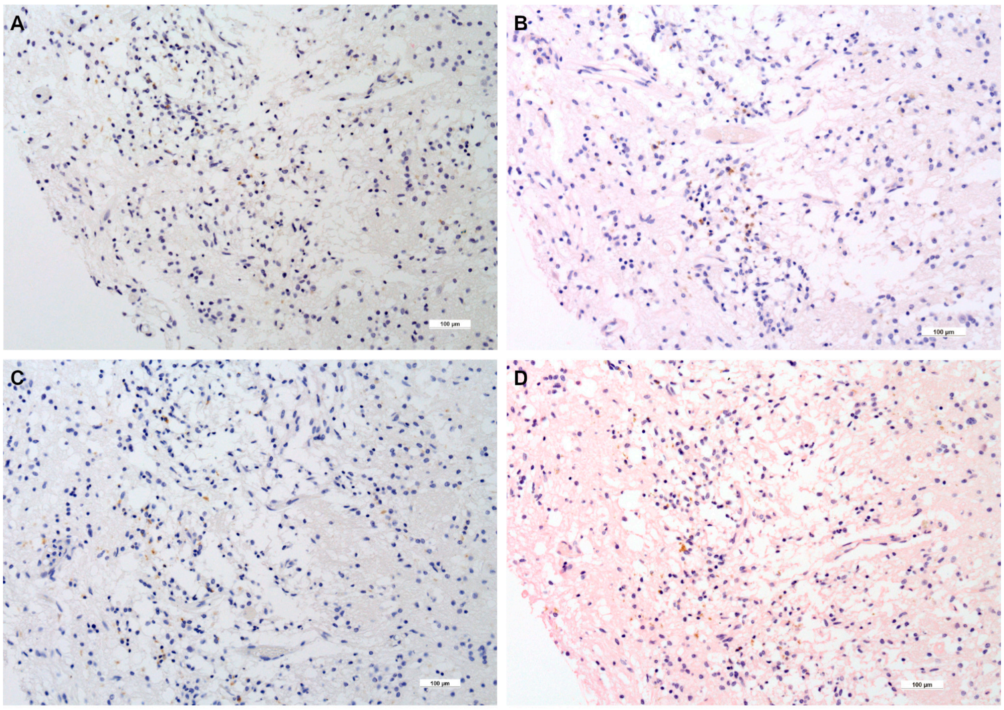

Figure S5: Negative controls for Figure 4, COVID⁺, immunohistochemical reactions in olfactory bulb tissue. Controls for: OMP reaction (**A**), CD3 reaction (**B**) CD8 reaction (**C**), and CD68 reaction (**D**). Scale bar 100 µm. Abbreviations: COVID⁻, control group; OMP, olfactory marker protein; CD3, marker for T cells; CD8, marker for cytotoxic T cells; CD68, marker for macrophages.

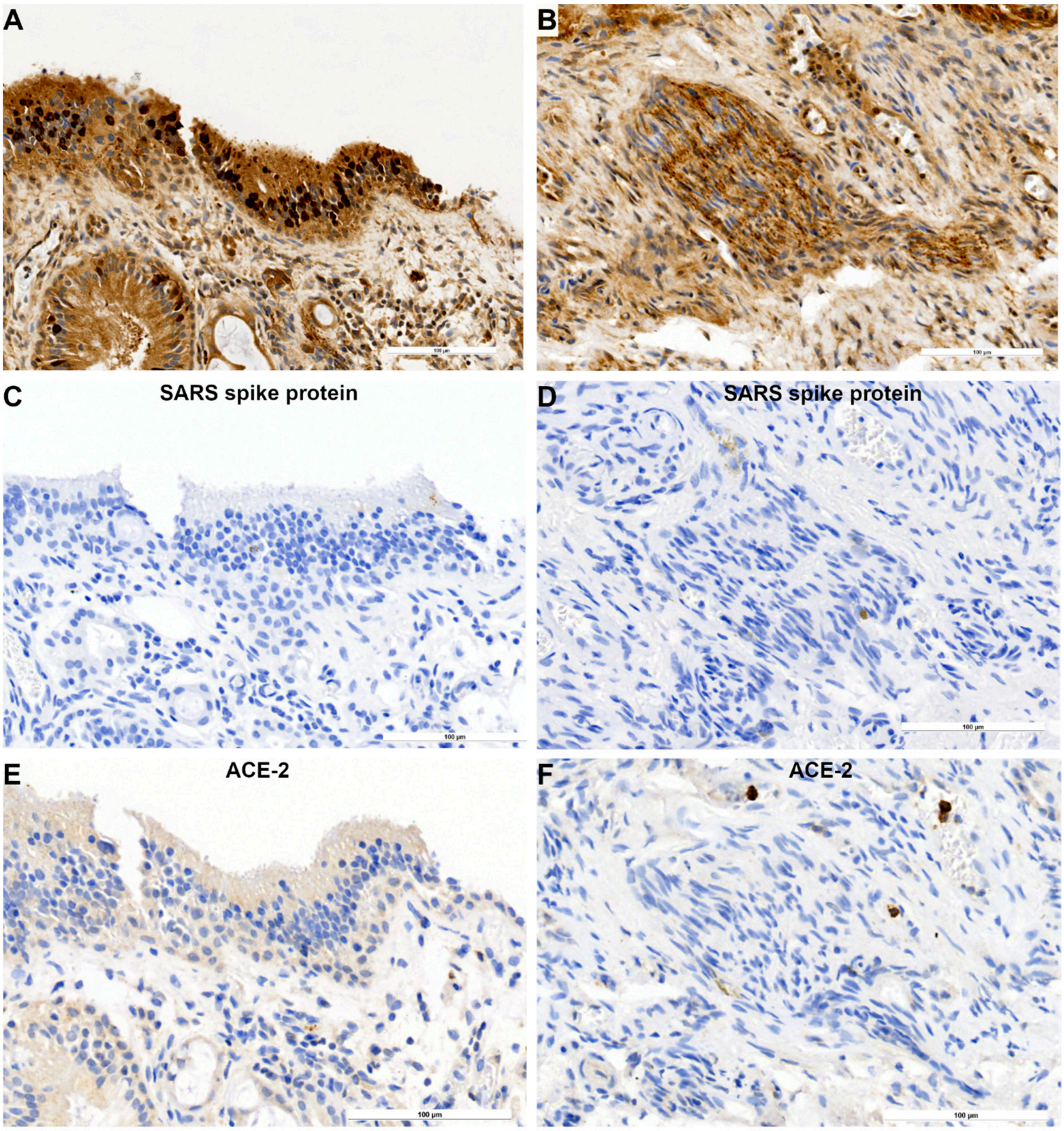

Figure S6: COVID+, Immunohistochemical reactions in OE (**A, C, E**) and olfactory nerves (**B, D, F**). This specimen reveals positive reactions for SARS spike protein in the OE (**C**) and in the olfactory nerve (**D**). ACE-2 reactions are restricted to blood vessels next to the olfactory nerve (**F**).Scale bar 100 µm.

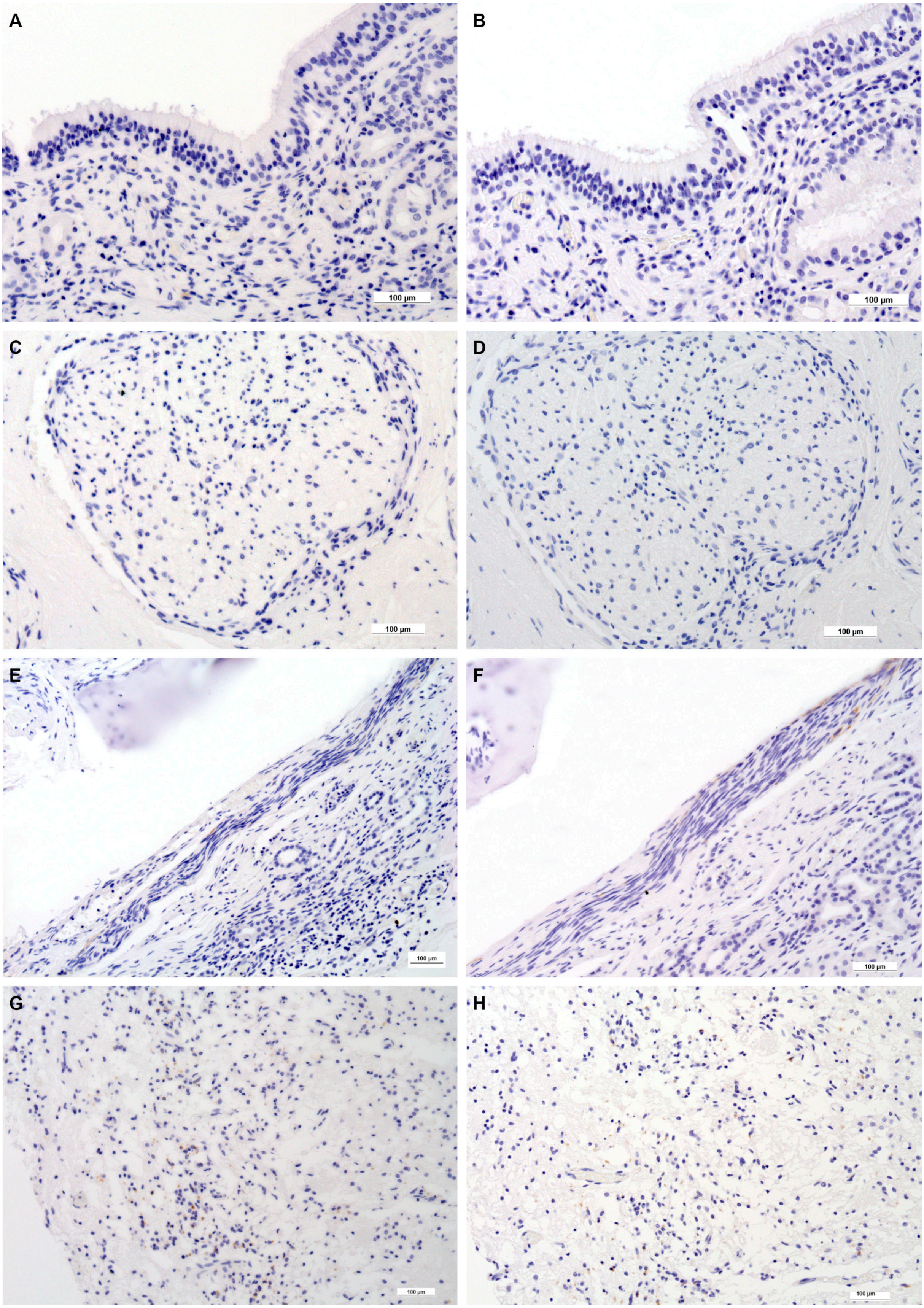

Figure S7: Negative controls for Figure 6, COVID$^+$, immunohistochemical reactions in different tissues. Controls for SARS spike protein reactions in epithelium (**A**), nerve tissues (**C,E**) and olfactory bulb tissue (**G**). Controls for ACE-2 reactions in epithelium (**B**), nerve tissues (**D,F**) and olfactory bulb tissue (**H**). Scale bar 100 µm. Abbreviations: COVID$^+$, patients with SARS-CoV-2 infection.

Protocol S9 Immunohistochemical reactions

1. Deparaffination 3x for 10 min in xylene, 5 min in xylene/ethanol mix in a ratio 1:1 followed by descending ethanol series (100 %, 96 %, 90 %, 80 %, 70 %) until distilled water for 3 min, respectively
2. Antigen retrieval in corresponding buffer (TRIS-EDTA, Carl Roth, Karlsruhe, Germany; Citrate, Merck and VWR Chemicals BDH, Darmstadt, Germany) in the microwave for 5 min at 600W and 5 min at 80W according Table 2. Afterwards, samples in the sample holder were cooled down with running water for 15 min
3. 3x washing step with 0.1 M PBS for 5 min (Gibco™ PBS-Tabletten, Fisher Scientific GmbH, Schwerte, Germany)
4. Peroxidase inhibition with 3 % H_2O_2 on a nutating mixer for 30 min at room temperature
5. 3x washing step with 0.1 M PBS for 5 min
6. Blocking of the non-specific binding sites 5 % normal goat serum in 0.1 M PBS in a wet chamber for 1 h at room temperature
7. Removal of NGS solution
8. According to Table 2, primary antibodies were diluted in 0.1 M PBS and 3 % normal goat serum. Incubation in a wet chamber overnight at 4°C
9. On the next day, 3x washing step with 0.1 M PBS for 5 min
10. Secondary antibodies were diluted 1 : 200 in 0.1 M PBS, 3 % normal goat and incubated in a wet chamber for 1 h at room temperature
11. Component A and component B from the avidin/biotin (AB-)complex by Vectastain was diluted 1:50 in 0.1 M PBS and incubated at least 30 min prior to use
12. 3x washing step with 0.1 M PBS for 5 min
13. Incubation of the AB-complex in a wet chamber for 1 h at room temperature
14. 3x washing step with 0.1 M PBS for 5 min
15. Preparation of DAB solution 1 : 50 in 0.1 M PBS
16. Incubation of DAB for 7 min at room temperature
17. Reaction stop with tap water
18. Washing step with distilled water for 5 min
19. Counterstaining with hemalaun for < 10 s and stopping the reaction with tap water
20. Dehydration with increasing ethonal series (70 %, 80 %, 90 %, 96 %, 100 %) for 3 min and 3x for 10 min in xylene
21. Mounting in DePeX and coverslipping
